# The Electrical and Thermal Characteristics of Stacked GaN MISHEMT

**DOI:** 10.3390/mi13122101

**Published:** 2022-11-28

**Authors:** Caixin Hui, Qiuqi Chen, Yijun Shi, Zhiyuan He, Yun Huang, Xiangjun Lu, Hongyue Wang, Jie Jiang, Guoguang Lu

**Affiliations:** 1 School of Materials Science and Engineering, Xiamen University of Technology, Xiamen 361024, China; 2 China Electronic Product Reliability and Environmental Testing Research Institute, Guangzhou 511370, China

**Keywords:** stacked GaN MISHEMTs, electrical performance parameters, thermal coupling, thermal resistance model

## Abstract

To study the working performance of 3D stacked chips, a double-layer stacked GaN MISHEMTs structure was designed to study the electro-thermal characteristics and heat transfer process of stacked chips. Firstly, the electrical characteristics of double-layer and single-layer GaN MISHEMTs are compared at room temperature. Under the same conditions, the output current of double-layer GaN MISHEMTs is twice that of single-layer GaN MISHEMTs, but its off-state current is much higher than that of a single-layer device. Meanwhile, there is no significant difference between the threshold voltages of the double-layer and single-layer GaN MISHEMTs. Then, the effect of temperature on the electrical characteristics of double-layer GaN MISHEMTs is also investigated. When the temperature increased from room temperature to 150 °C, the device’s threshold voltage gradually shifted negatively, the output current of the device decreased, and the off-state current of the device increased. Furthermore, a thermal resistance network model has been established to analyze the thermal characteristics of the stacked GaN MISHEMTs. The relative error between the results calculated according to the model and the experimental results does not exceed 4.26%, which verified the correctness and accuracy of the presented model to predict the temperature distribution of the stacked GaN MISHEMTs.

## 1. Introduction

By virtue of their wide energy bandgap, high electron saturation velocity, and especially the polarization-induced high density and high mobility 2-dimension electron gases (2DEG), GaN-based devices are becoming increasingly popular in power applications that require high breakdown voltage, high temperature operating capability, and high-power conversion efficiency [1,2,3,4]. GaN-based metal insulator semiconductor high electron mobility transistors (MISHEMTs) have especially attracted much attention due to their large effective gate swing (e.g., >10 V) and significantly reduced the gate leakage current [3,4,5,6]. To improve the GaN-based power system’s working frequency and power density, reducing the parasitic parameters of the power system is an effective method. The current commonly adopted method is to integrate one or more GaN power devices with corresponding drive circuits and protection circuits on a single chip (system on chip, SoC) or in the same package (system in package, SiP [7]), which is divided into planar package integration and three-dimensional stacked package integration. The three-dimensional integrated GaN power system features multiple GaN power devices and corresponding control chips in a single package through through-silicon-via (TSV), a redistribution layer (RDL), wire bonding, or other advanced three-dimensional packaging methods [8]. It can be inferred that the 3D-integrated GaN power system has many advantages such as high performance and miniaturization, which makes it ideal for the research directions of GaN power systems. However, both the reduction in the system volume and the increase in the working frequency and power density will lead to a more significant electrothermal coupling effect on the 3D-integrated GaN power system, which will cause a sharp increase in heat accumulation and may eventually lead to a significant rise in the temperature of the devices [9].

In this work, the electrical and thermal characteristics of the stacked GaN MISHEMT are analyzed. This research is arranged as follows. The structure of the double-layer GaN MISHEMT and experimental setups are introduced In Section 2. In Section 3, at first, the electrical characteristics of the double-layer and single-layer GaN MISHEMTs are compared. Then, the effect of temperature on the electrical characteristics of double-layer GaN MISHEMT is also investigated. Furthermore, a thermal resistance network model has been established to analyze the thermal characteristics of the stacked GaN MISHEMT. Finally, the conclusion is summarized in Section 4.

## 2. Structure and Experiment

**Figure 1:**
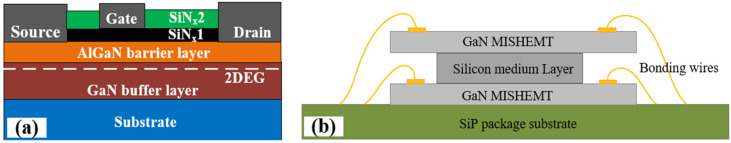
(**a**) Schematic device structure of GaN MISHEMT, (**b**) double-layer GaN MISHEMT.

**Figure 2:**
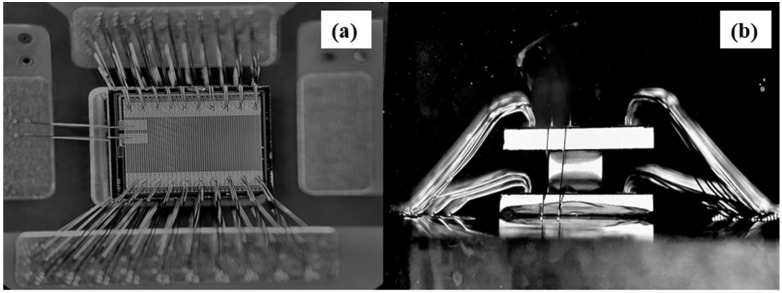
(**a**) Top view of stacked GaN MISHEMT, (**b**) side view of stacked GaN MISHEMT.

The schematic device structure of GaN MISHEMT is shown in Figure 1a, and the stacking scheme shown in Figure 1b is employed for double-layer GaN MISHEMT. In this stacking scheme, single crystal silicon (semi-insulated silicon) is selected as the intermediate medium and fixed between the two MISHEMTs by insulating adhesive (Major component: Phenolic epoxy resin). This structure makes the top layer chip suspended, which is not only conducive to wires bonding, but can also replace the silicon medium with a corresponding drive circuit and protection circuit and enrich the device design. The material object of the stacked two-layer MISHEMT is shown in Figure 2. As shown, the electrodes of two GaN MISHEMTs are bonded to the same pad on the substrate [10].

**Figure 3:**
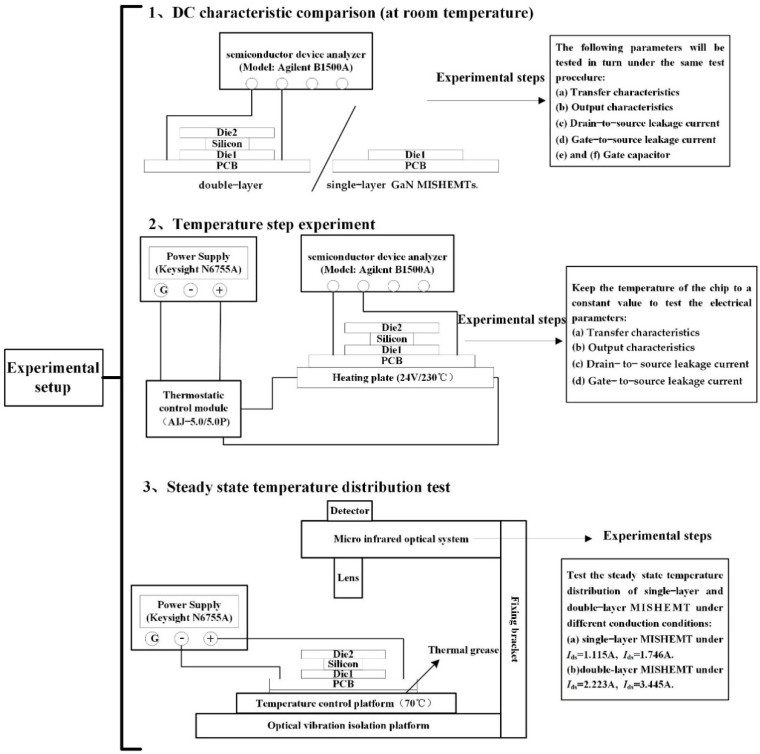
Experimental setup diagram.

The experiment was mainly divided into three parts: (1) The electrical characteristics of single-layer and double-layer GaN MISHEMTs were measured and compared by the semiconductor device analyzer (Model: Agilent B1500A is produced by Keysight, Santa Rosa, CA, USA) at room temperature; (2) The effect of temperature on the electrical characteristics of double-layer GaN MISHEMT is also investigated with the temperature changed from room temperature to 150 °C; (3) A thermal resistance network model [11] has been established to analyze the thermal characteristics of the stacked GaN MISHEMT. Finally, the DC power (Keysight N6755A, Santa Rosa, CA, USA) will apply power to the MISHEMTs, and the steady state surface temperatures of MISHEMTs will be detected by the high-speed dynamic infrared thermal imager (model: TM-HST is produced by QFI, USA) to verify the correctness of the thermal resistance network model. The experimental setups are shown in Figure 3.

## 3. Results and Discussion

A. The electrical characteristics of the double-layer GaN MISHEMT

**Figure 4:**
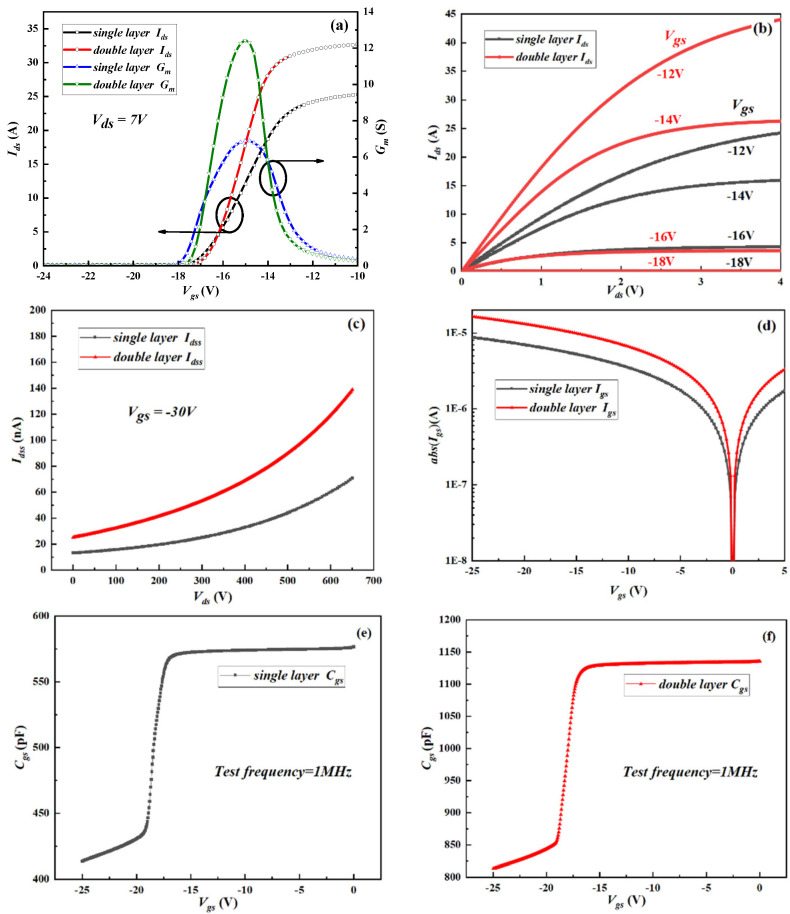
The comparison between the electrical properties of the single−layer and double−layer GaN MISHEMTs. (**a**) Transfer characteristics, (**b**) output characteristics, (**c**) drain−to−source leakage current, (**d**) gate−to−source leakage current, (**e**,**f**) gate capacitor. (Data was measured by the semiconductor device analyzer (Agilent B1500 and B1505 are produced by Keysight, Santa Rosa, CA, USA).

Figure 4 shows the comparison between the electrical properties of the single-layer and double-layer GaN MISHEMTs. As shown in Figure 4a,b, the output current (*I*_ds_) of the stacked GaN MISHEMT is twice that of the single-layer device. Likewise, there is no significant difference between the threshold voltages (*V*_th_) of the double-layer and single-layer GaN MISHEMTs. The increase of *I*_ds_ is beneficial to reduce the power consumption of the device and improve the efficiency of the system. Figure 4c,d exhibit the drain-to-source leakage current (*I*_dss_) and gate-to-source leakage current (*I*_gs_) of the single-layer and double-layer GaN MISHEMTs. As expected, the drain-to-source leakage current and gate-to-source leakage current of the stacked GaN MISHEMT is also higher than that of the single-layer device. With a drain voltage (*V*_ds_) of 650 V, the drain-to-source leakage current of the single-layer MISHEMT is 73.5 nA, which is about half of the drain-to-source leakage current (*I*_dss_ = 140 nA) of the double-layer MISHEMT. The increase in the drain-to-source leakage current and gate-to-source leakage current will increase the power consumption in the off state [12]. The gate-to-source capacitances (*C_gs_*) of the single-layer and double-layer GaN MISHEMTs are plotted in Figure 4e,f. As shown, the gate-to-source capacitance of the double-layer MISHEMT is also twice of that of the single-layer device. At the same time, the steep slope trend of the curve is almost unchanged. When the gate of the GaN MISHEMT is turned on, the gate capacitor will not change and remains stable [13,14].

**Figure 5:**
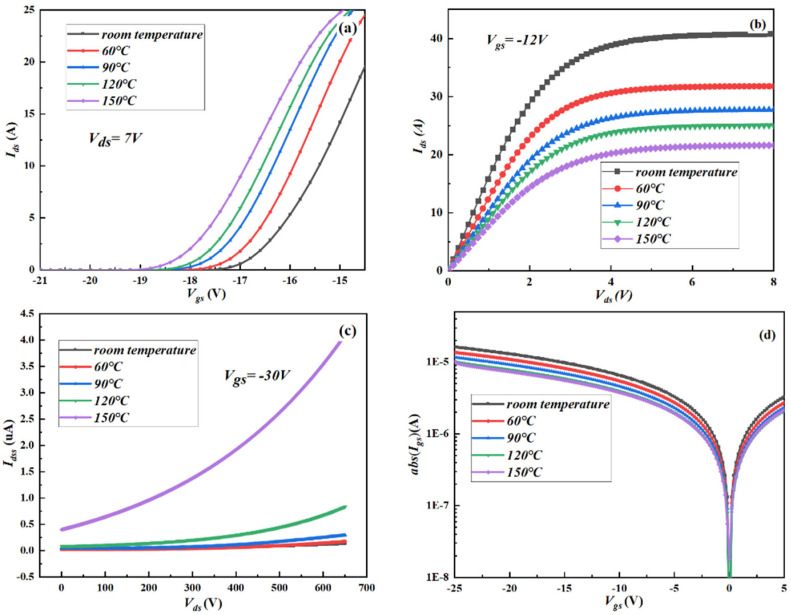
The influence of the temperature on the electrical properties of the double−layer GaN MISHEMTs. (**a**) Transfer characteristics, (**b**) output characteristics, (**c**) drain−to−source leakage current, (**d**) gate−to−source leakage current. Data was measured by the semiconductor device analyzer (Agilent B1500 and B1505 are produced by Keysight, Santa Rosa, CA, USA).

The influence of temperature on the electrical properties of the double-layer GaN MISHEMT is also investigated, as shown in Figure 5. As it can be seen in Figure 5a, the threshold voltage of stacked MISHEM in the bias state (*V_ds_* = 7 V) gradually shifts negatively as the temperature increased. When the temperature is increased from room temperature to 150 °C, the threshold voltages of the stacked GaN MISHEMT are reduced from −16.31 V to −17.94 V, which is attributable to the detrapping effect in the insulating oxide layer, AlGaN barrier layer, and the oxide/AlGaN interface [15]. The electron detrapped from the electron traps decreases negative charges in the gate region and subsequently decreases the device’s threshold voltage. Figure 5b shows the effect of temperature on the output current (*I_ds_*) of the double-layer GaN MISHEMT under the bias state (*V_gs_* = −12 V). The output current of the double-layer GaN MISHEMT is decreased when the temperature increases. When the temperature is increased from room temperature to 150 °C, the output current of the double-layer GaN MISHEMT is decreased by about 10 A, which is due to the increase in temperature that will lead to a significant decrease in the electron mobility. The influence of the temperature on the drain-to-source leakage current at the bias states of (*V_gs_* = −30 V) and gate-to-source leakage current (*I*_gs_) as *V_gs_* is scanned from −25 V to 5 V of the double-layer GaN MISHEMT is also analyzed, as shown in Figure 5c,d. It can be seen that the drain-to-source leakage current of the double-layer GaN MISHEMT is increased when the temperature increases. When the temperature is increased from room temperature to 150 °C, the drain-to-source leakage current of the double-layer GaN MISHEMT is increased from 0.17 μA to 4 μA, which may be due to the increase of the leakage current in the buffer layer; the gate-to-source leakage current of the double-layer GaN MISHEMT decreases when the temperature increases. When the temperature is increased from room temperature to 150 °C, the gate-to-source leakage current of the double-layer GaN MISHEMT is decreased from 16.5 μA to 10.2 μA, which may be due to the high temperature, which will decrease the average free path of the electron and thus increase the vibration energy of phonons and collision probability of electrons, leading to the reduction in the tunneling probability of electrons [16].

B. The thermal characteristics of the double-layer GaN MISHEMT

**Figure 6:**
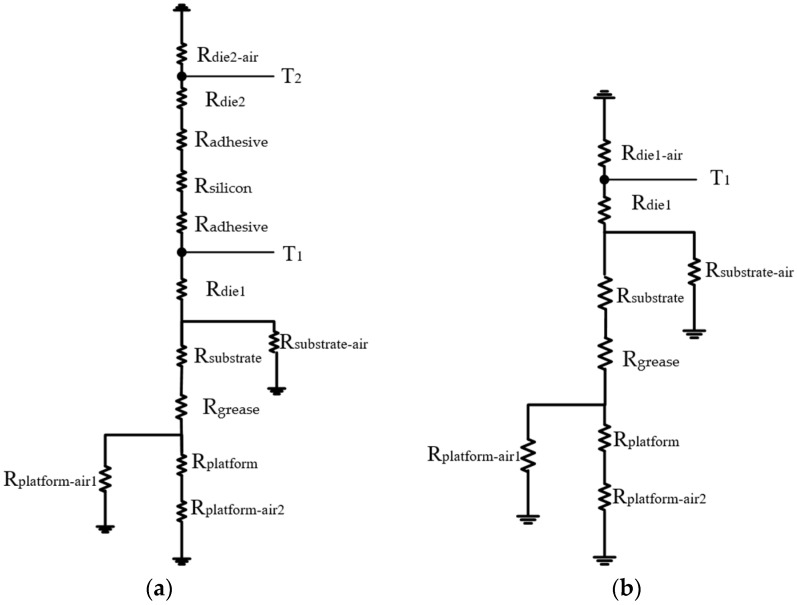
Thermal resistance network model of the single-layer and double-layer GaN MISHEMTs. (**a**) Double-layer device thermal resistance network, (**b**) Single-layer device thermal resistance network.

In this part, the steady-state temperature distribution of the devices will be tested. According to the actual test, a thermal resistance network model will be established to analyze the heat transfer path of double-layer GaN MISHEMT. According to the solid heat transfer theory, the main ways of heat transfer are conduction and convection. When devices are turned on, a part of the heat in the channel is transferred upward to the device’s surface and the other part of the heat is transferred downward to the substrate. Combined with the double-layer GaN MISHEMT’s test environment and platform, the thermal resistance network model of the double-layer GaN MISHEMT [17,18,19,20] is proposed (Figure 6a).

The thermal resistance of each part of the double-layer GaN MISHEMT will be calculated. According to the thermal resistance property formula of the material:

(1) $$R_{cond}=\frac{L}{A\times K}$$

where *L* and *A* are the thickness and surface area of each part of the material, respectively; *K* is the thermal conductivity of the material, *R_cond_* is the thermal resistance of the material. The calculation formula of convection thermal resistance obtained from Newton’s law of cooling is:

(2) $$R_{conv}=\frac{1}{A\times h}$$

where *h* and *A* are the air convective heat transfer coefficient and material surface area, respectively; *R_conv_* is the convective thermal resistance. Due to the stacked structure of the double-layer GaN MISHEMT, the heat transfer path will be from the narrow-area die to the wide-section substrate. Therefore, the thermal resistance calculation method of 45° will be used to calculate the steady-state thermal resistance value. Through Equations (1) and (2) and the thermal resistance calculation method of 45°, the thermal resistances of each part of the device are calculated as shown in Table 1.

As shown in the thermal resistance network model in Figure 6a, the heat generated by the die1 of the double-layer GaN MISHEMT has two mainly paths for heat dissipation. The thermal resistances on the two paths are connected in parallel. The thermal resistance from die1 to the air (*R*_T1−air_) can be calculated as follows [18]:

(3) $$\begin{array}{l} R_{T1-\mathrm{air}}=(R_{die2-\mathrm{air}}+R_{die2}+R_{silicon}+2\times R_{adhesive})//\{R_{die1}+R_{substrate-air}//[R_{substrate}\\ +R_{grease}+R_{platform-air1}//(R_{platform}+R_{platform-air2})]\} \end{array}$$

The calculation equation of the thermal resistance from the upper chip die2 to the air (*R*_T2−_air__) is as follows:

(4) $$\begin{array}{l} R_{T2-\mathrm{air}}=R_{die2-\mathrm{air}}//\{R_{die2}+R_{silicon}+2\times R_{adhesive}+R_{die1}+R_{substrate-air}//[R_{substrate}\\ +R_{grease}+R_{platform-air1}//(R_{platform}+R_{platform-air2})]\} \end{array}$$

The calculation method of the thermal resistance from the single-layer chip to the air in Figure 6b can be obtained by removing *R*_die1_, *R*_silicon_, and 2 × *R*_adhesive_ from the Equation (4). According to the above equation, *R*_T1−air_ is 66.80 °C/W for the single-layer MISHEMT. For the double-layer MISHEMT, *R*_T1−air_ and *R*_T2−air_ are 67.05 °C/W and 90.41 °C/W, respectively. When multiple chips are turned on at the same time, there is a temperature coupling phenomenon [19,20,21]. The thermal resistance of the bottom test platform is small, thus the heat dissipation is fast and most of the heat spreads downward. Therefore, the bottom chip will be superimposed by the heat of the top chip. The calculation equation to calculate the surface temperature is as follows [19]:

(5) $$T_{i}=(\sum_{k=1}^{n}P_{k})\times R_{T1-\mathrm{air}}+(\sum_{k=2}^{n}P_{k})\times r_{2}+\ldots +(\sum_{k=i}^{n}P_{k})\times r_{i}+T_{air}$$

where i is the number of chip layers to be calculated, *n* is the total number of stacked chips; *T_air_* is room temperature. In this work, the initial value (70 °C) of the temperature control platform was selected as *T_air_*. *P_k_* is the power value applied by layer k chip, *r_i_* is the difference between the adjacent chips’ thermal resistances to air (e.g., *r*_1_ = *R_T_*_2−*air*_ *− R_T_*_1−*air*_), which can calculate the temperature of each layer of chips or the temperature difference between each layer of chips when multiple chips work at the same time according to Equation (5).

**Figure 7:**
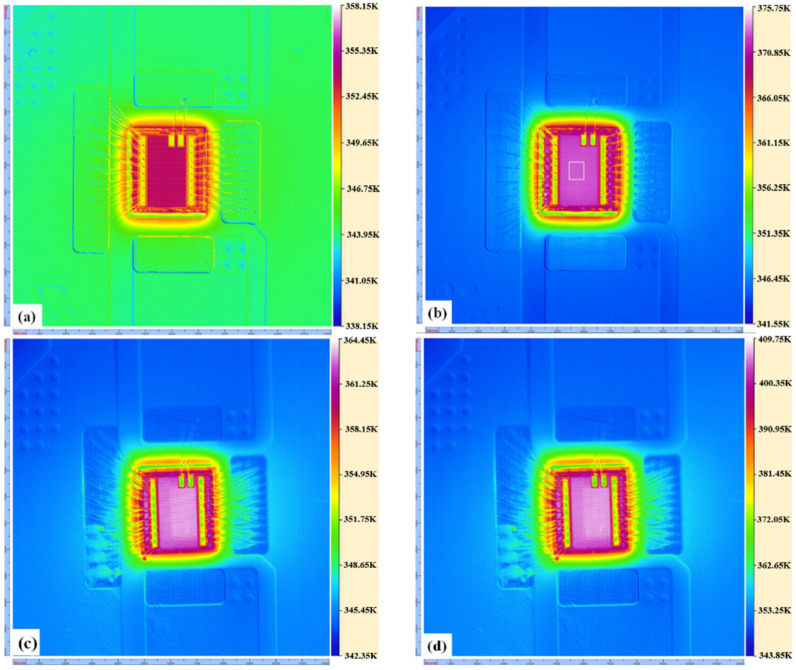
Temperature distribution of devices under different *I*_ds_: (**a**) single−layer MISHEMT under *I*_ds_ = 1.115 A, (**b**) single−layer MISHEMT under *I*_ds_ = 1.746 A, (**c**) double−layer MISHEMT under *I*_ds_ = 2.223 A, (**d**) double−layer MISHEMT under *I*_ds_ = 3.445 A. (The steady−state surface temperatures of MISHEMTs are detected by the high−speed dynamic infrared thermal imager (TM−HST)).

To verify the correctness of the thermal resistance models, we have measured the surface temperatures of the devices under different currents, as shown in Figure 7. Since the thermal imaging camera can only measure the surface temperature of the top chip, the surface temperature of the top chip of the single-layer and double-layer devices were calculated and compared according to Equation (5). The comparison results and errors are shown in Table 2. The results show that when the total current flowing through the double-layer MISHEMT is twice that of the single-layer MISHEMT, the temperature rise of the double-layer MISHEMT is nearly twice that of the single-layer MISHEMT.

According to the data comparison in Table 2, the maximum relative error between the calculated value and the measured value of the device surface temperature under different input currents does not exceed 4.62%. The calculated results are well supported by the experimental measurements, which verified that the established thermal resistance network models of single-layer and double-layer stacked GaN MISHEMTs are basically correct.

## 4. Conclusions

This work presents the electrical and thermal characteristics of the stacked GaN MISHEMT. As expected, the output current of the stacked GaN MISHEMT is twice that of the single-layer device, but its off-state current is much higher than that of the single-layer device. Meanwhile, there is no significant difference between the threshold voltages of the double-layer and single-layer GaN MISHEMTs. The effect of temperature on the electrical characteristics of double-layer GaN MISHEMT is also investigated. We found that when the temperature increased from room temperature to 150 °C, the device’s threshold voltage gradually shifts negatively, and there is a decrease in device’s output current and an increase in its off-state current. These phenomena are similar to that the single-layer GaN MISHEMT. Furthermore, a thermal resistance network model has been established to analyze the thermal characteristics of the stacked GaN MISHEMT. The calculated results are well supported by the experimental measurements, which verified the correctness and accuracy of the presented model to predict the temperature distribution of the stacked GaN MISHEMT. At present, chip stacking is an effective way to improve the power processing capacity. The research in this paper investigates the electrothermal characteristics and temperature prediction model of the stacked GaN MISHEMT, which can provide a reference for the design and application of the stacked GaN MISHEMT. However, the integration process required for chip stacking is more complex than that of the planar integration, and the reduction of the interconnection distance between chips will also cause thermal coupling problems.

## Figures and Tables

**Table 1:** Calculated values of thermal resistance of each part of the device.

Thermal Resistance	Calculated Value [°C/W]	Thermal Resistance	Calculated Value [°C/W]
*R*_die−air_	10,775.86	*R*_die1_ = *R*_die2_	0.25
*R*_substrate_	111.11	*R*_substrate−air_	162.41
*R*_platform_	0.05	*R*_platform−air1_	7.26
*R*_platform−air2_	6.94	*R*_silicon_	1.90
*R_adhesive_*	10.78	*R_thermal grease_*	0.03

**Table 2:** Comparisons: measured result and calculated result of surface temperature.

Test Devices	Input Current (A)	Dissipation Power (W)	Measured Temperature (K)	Calculated Temperature (K)	Relative Error (%)
single-layer MISHEMT	1.115	0.155	354.75	353.50	0.35
	1.746	0.455	373.85	373.54	0.08
double-layer MISHEMT	2.223	0.320	364.45	372.08	2.09
	3.445	0.938	409.05	427.95	4.62

## Data Availability

Data available on request due to restrictions, e.g., privacy or ethical.
